# Supplementary material for: Unbiased high-throughput characterization of mussel transcriptomic responses to sublethal concentrations of the biotoxin okadaic acid
Source: PeerJ. 2015 Nov 19;3:e1429. doi: 10.7717/peerj.1429 (PMC4655091; doi:10.7717/peerj.1429)
Supplement: Supplemental Information 1 [file peerj-03-1429-s001.docx]

Supplementary Material S1. List of differentially expressed transcripts with annotation found in digestive gland tissue showing an expression change greater than 100-fold (|logFC| > 2) in the microarray analysis.

| UPREGULATED | |
| --- | --- |
| **description** | **logFC** |
| nose resistant to fluoxetine protein 6 | 8.95 |
| autocrine motility factor receptor | 8.80 |
| c1q domain containing protein 1q3 | 8.77 |
| hypothetical protein CGI_10026086 [Crassostrea gigas] | 7.86 |
| cathepsin d | 7.55 |
| nacre protein | 6.74 |
| bcl2 adenovirus e1b 19-kd protein-interacting | 6.60 |
| proteasome beta 4 subunit | 6.58 |
| mytimacin-5 partial | 6.42 |
| endo-1,3-beta-xylanase | 6.38 |
| c1q domain containing protein 1q25 | 6.36 |
| cathepsin b | 5.83 |
| mantle gene 8 | 5.32 |
| collagen alpha-5 chain | 5.11 |
| interferon-inducible gtpase 5-like | 5.10 |
| acetylcholinesterase | 4.96 |
| hypothetical protein CGI_10012557 [Crassostrea gigas] | 4.72 |
| deleted in malignant brain tumors 1 | 4.64 |
| atpase h+ transporting lysosomal 21 kda v0 subunit | 4.50 |
| superoxide dismutase | 4.42 |
| vitelline membrane outer layer protein 1 homolog | 4.38 |
| col protein | 4.37 |
| uncharacterized protein loc102449188 | 4.24 |
| hypothetical protein CGI_10015342 [Crassostrea gigas] | 4.24 |
| proteasome subunit beta type-4 | 4.09 |
| developmentally-regulated vdg3 | 3.98 |
| cell adhesion molecule-related down-regulated by oncogenes-like | 3.92 |
| vdg3 [Mytilus edulis] | 3.87 |
| neurogenic locus notch homolog protein 2-like | 3.85 |
| transitional endoplasmic reticulum atpase | 3.85 |
| peptidylglycine alpha-amidating monooxygenase precursor | 3.79 |
| collagen alpha-4 chain | 3.78 |
| fibrinogen c domain-containing protein 1 | 3.76 |
| c-binding protein | 3.75 |
| fibrinogen-like protein a | 3.71 |
| ankyrin repeat domain-containing protein 50 | 3.68 |
| mytimycin precursor | 3.67 |
| gtpase imap family member 7-like | 3.65 |
| hypothetical protein CGI_10011963 [Crassostrea gigas] | 3.58 |
| cytoplasmic partial | 3.54 |
| uncharacterized protein loc101862413 | 3.53 |
| kazal-like serine protease inhibitor domain-containing protein | 3.53 |
| type-2 ice-structuring | 3.51 |
| nidogen and egf-like domain-containing protein 1 | 3.49 |
| hypothetical protein [Paramecium tetraurelia strain d4-2] | 3.49 |
| gtpase imap family member 4-like | 3.48 |
| hypothetical protein [Acinetobacter sp. ANC 3789] | 3.42 |
| peptidylglycine alpha-amidating monooxygenase | 3.35 |
| sec1 family domain-containing protein 2 | 3.35 |
| fimbrial protein pilin | 3.34 |
| hypothetical protein CGI_10003274 [Crassostrea gigas] | 3.34 |
| polyubiquitin | 3.31 |
| synaptosomal-associated protein 25 | 3.25 |
| proline iminopeptidase | 3.19 |
| heavy metal-binding protein hip | 3.17 |
| uncharacterized protein loc101850813 | 3.14 |
| jagged protein | 3.13 |
| apical endosomal glyco | 3.11 |
| low-density lipoprotein receptor-related protein 6 | 3.02 |
| nc domain-containing protein | 3.02 |
| component of the counting factor complex | 3.00 |
| hypothetical protein CGI_10020658 [Crassostrea gigas] | 2.99 |
| nfx1-type zinc finger-containing protein 1 | 2.99 |
| apoptosis inhibitor iap | 2.99 |
| mammalian ependymin-related protein 1 | 2.98 |
| si:ch211- protein | 2.94 |
| hypothetical protein BRAFLDRAFT_106560 [Branchiostoma floridae] | 2.91 |
| nattectin precursor | 2.89 |
| hypothetical protein | 2.88 |
| hypothetical protein CGI_10012644 [Crassostrea gigas] | 2.86 |
| endo-1,3-beta-d-glucanase | 2.85 |
| peroxisomal proliferator-activated receptor a-interacting complex 285 kda | 2.83 |
| fibrinogen-related protein | 2.83 |
| rab gdp dissociation inhibitor beta | 2.79 |
| nadh dehydrogenase subunit 6 | 2.79 |
| pancreatic secretory granule membrane major glycoprotein gp2 | 2.78 |
| peptidyl-glycine alpha-amidating monooxygenase-like | 2.70 |
| oncoprotein-induced transcript 3 protein | 2.66 |
| heat shock 70 kda protein 12b | 2.66 |
| daz interacting protein zinc finger-like | 2.65 |
| complement c1q tumor necrosis factor-related protein 3 | 2.60 |
| neuronal nitric oxidse synthase protein | 2.59 |
| uncharacterized protein loc580197 | 2.59 |
| hypothetical protein BRAFLDRAFT_79532 [Branchiostoma floridae] | 2.59 |
| predicted protein [Nematostella vectensis] | 2.58 |
| 60 kda ss-a ro ribonucleoprotein | 2.53 |
| propionyl- carboxylase alpha mitochondrial | 2.53 |
| succinate dehydrogenase | 2.51 |
| hypothetical protein DAPPUDRAFT_255671 [Daphnia pulex] | 2.50 |
| elongation factor 2 | 2.47 |
| sialic acid binding lectin | 2.47 |
| syringomycin biosynthesis enzyme | 2.42 |
| protocadherin fat 4-like | 2.41 |
| phenylalanine hydroxylase | 2.40 |
| PREDICTED: neuroglian-like [Acyrthosiphon pisum] | 2.38 |
| hypothetical protein CGI_10013901 [Crassostrea gigas] | 2.36 |
| low affinity immunoglobulin epsilon fc receptor | 2.35 |
| vitelline membrane outer layer protein 1 | 2.34 |
| group xvi phospholipase a2 | 2.32 |
| gtpase imap family member 4 | 2.28 |
| hypothetical protein CGI_10014841 [Crassostrea gigas] | 2.26 |
| gtpase imap family member 8-like | 2.26 |
| uncharacterized protein loc585517 isoform 2 | 2.25 |
| translational elongation factor-2 | 2.19 |
| virion core protein (lumpy skin disease virus) | 2.19 |
| tartrate-resistant acid phosphatase type 5 | 2.17 |
| complement c1q-like protein 4 | 2.12 |
| endoglucanase [Mizuhopecten yessoensis] | 2.11 |
| interferon alpha-inducible protein 27-like protein 2-like | 2.09 |
| complement c1q tumor necrosis factor-related protein 8 | 2.09 |
| microtubule-associated protein futsch | 2.08 |
| Titin [Crassostrea gigas] | 2.05 |
| ankyrin unc44 | 2.05 |
| jagged 1-like | 2.04 |
| 3-hydroxyisobutyrate mitochondrial-like | 2.03 |
| hypothetical protein CGI_10008425 [Crassostrea gigas] | 2.01 |
|  |  |
|  |  |
| DOWNREGULATED | |
| **description** | **logFC** |
| sodium-dependent neutral amino acid transporter b at2-like | -2.01 |
| alpha-tubulin | -2.04 |
| ribonucleoside-diphosphate reductase subunit m2-like | -2.05 |
| ribonucleotide reductase m2 polypeptide | -2.06 |
| alpha-1,3-mannosyl-glycoprotein 4-beta-n-acetylglucosaminyltransferase b | -2.10 |
| kex2p | -2.13 |
| gastrointestinal growth factor xp4-like | -2.14 |
| vitelline envelope zona pellucida domain protein 14 | -2.26 |
| kif21a protein | -2.29 |
| reticulon-like protein | -2.30 |
| phosphatidylinositol-binding clathrin assembly | -2.31 |
| low quality protein: solute carrier organic anion transporter family member 5a1 | -2.39 |
| zona pellucida domain-containing protein 1 | -2.41 |
| conserved protein | -2.47 |
| cytokine induced apoptosis inhibitor 1 | -2.62 |
| hypothetical protein LOTGIDRAFT_155380 [Lottia gigantea] | -2.71 |
| hypothetical protein [Vibrio anguillarum] | -2.78 |
| schlafen family member 13 | -2.88 |
| hypothetical protein CGI_10008221 [Crassostrea gigas] | -3.04 |
| microsomal glutathione s-transferase 3-like protein | -3.21 |
| dna-directed rna polymerase | -3.46 |
| starch-binding domain-containing protein 1 | -3.48 |
| hypothetical protein [Butyricimonas synergistica] | -3.77 |
| peroxisomal acyl-coenzyme a oxidase 2 | -4.18 |
| probable small nuclear ribonucleoprotein sm d2-like | -5.05 |
| achain reduced peptidylglycine alpha-hydroxylating monooxygenase | -5.76 |
