# Supplementary material for: Unbiased high-throughput characterization of mussel transcriptomic responses to sublethal concentrations of the biotoxin okadaic acid
Source: PeerJ. 2015 Nov 19;3:e1429. doi: 10.7717/peerj.1429 (PMC4655091; doi:10.7717/peerj.1429)
Supplement: Supplemental Information 2 [file peerj-03-1429-s002.docx]

Supplementary Material S2. List of differentially expressed transcripts with annotation found in gill tissue showing an expression change greater than 100-fold (|logFC| > 2) in the microarray analysis.

| UPREGULATED | |
| --- | --- |
| **description** | **logFC** |
| actin type-1 partial [Ostrea edulis] | 4.52 |
| mytimacin- partial | 3.47 |
| hypothetical protein CGI_10003274 [Crassostrea gigas] | 2.47 |
| bcl2 adenovirus e1b 19-kd protein-interacting | 2.43 |
| hypothetical protein CGI_10026086 [Crassostrea gigas] | 2.27 |
| vitelline envelope zona pellucida domain 9 | 2.09 |
| c-type lectin domain family 4 member g | 2.03 |
|  |  |
|  |  |
| DOWNREGULATED | |
| **description** | **logFC** |
| rna-binding protein | -2.08 |
| probable small nuclear ribonucleoprotein sm d2-like | -2.25 |
| histone 3-like | -2.25 |
| apextrin-like protein | -2.72 |
| kif21a protein | -2.87 |
| vitelline membrane outer layer protein 1 homolog | -3.59 |
