# Supplementary material for: Unbiased high-throughput characterization of mussel transcriptomic responses to sublethal concentrations of the biotoxin okadaic acid
Source: PeerJ. 2015 Nov 19;3:e1429. doi: 10.7717/peerj.1429 (PMC4655091; doi:10.7717/peerj.1429)
Supplement: Supplemental Information 3 [file peerj-03-1429-s003.docx]

Supplementary Material S3. List of active metabolic pathways associated with upregulated enzymes in digestive gland identified by microarray analysis and mapped to KEGG database.

| **Pathway** |
| --- |
| Biosynthesis of antibiotics |
| Purine metabolism |
| Cysteine and methionine metabolism |
| Glutathione metabolism |
| Fatty acid degradation |
| Methane metabolism |
| Arginine and proline metabolism |
| Glycolysis / Gluconeogenesis |
| Drug metabolism - cytochrome P450 |
| Drug metabolism - other enzymes |
| beta-Alanine metabolism |
| Metabolism of xenobiotics by cytochrome P450 |
| alpha-Linolenic acid metabolism |
| Chloroalkane and chloroalkene degradation |
| Glycine, serine and threonine metabolism |
| One carbon pool by folate |
| Oxidative phosphorylation |
| Pyruvate metabolism |
| Valine, leucine and isoleucine degradation |
| Starch and sucrose metabolism |
| Tyrosine metabolism |
| Amino sugar and nucleotide sugar metabolism |
| Carbon fixation pathways in prokaryotes |
| Drug metabolism - cytochrome P451 |
| Metabolism of xenobiotics by cytochrome P451 |
| Drug metabolism - cytochrome P452 |
| Glycerophospholipid metabolism |
| Monobactam biosynthesis |
| Mucin type O-Glycan biosynthesis |
| Nicotinate and nicotinamide metabolism |
| Pantothenate and CoA biosynthesis |
| Phenylalanine, tyrosine and tryptophan biosynthesis |
| Sulfur metabolism |
| Tetracycline biosynthesis |
| Arginine biosynthesis |
| Biosynthesis of ansamycins |
| C5-Branched dibasic acid metabolism |
| Caffeine metabolism |
| Dioxin degradation |
| Folate biosynthesis |
| Glycosaminoglycan degradation |
| Glycosphingolipid biosynthesis - globo series |
| Inositol phosphate metabolism |
| Isoquinoline alkaloid biosynthesis |
| N-Glycan biosynthesis |
| Nitrogen metabolism |
| Other glycan degradation |
| Phosphatidylinositol signaling system |
| Primary bile acid biosynthesis |
| Riboflavin metabolism |
| Steroid degradation |
| Synthesis and degradation of ketone bodies |
| Taurine and hypotaurine metabolism |
| Thiamine metabolism |
| Various types of N-glycan biosynthesis |
| Xylene degradation |
